# The State of Microbiological Cleanliness of Surfaces and Equipment of an Endoscopic Examination Laboratory—Data from a Reference Tertiary Clinical Endoscopy Center in Southern Poland

**DOI:** 10.3390/ijerph18126346

**Published:** 2021-06-11

**Authors:** Jolanta Gruszecka, Rafał Filip, Dorota Gutkowska

**Affiliations:** 1 Institute of Health Sciences, Medical College of Rzeszow University, 35-310 Rzeszow, Poland; gutkowska@ur.edu.pl; 2 Department of Clinical Microbiology, Clinical Hospital No. 2 im. Św. Jadwigi Królowej, 35-301 Rzeszow, Poland; 3 Faculty of Medicine, Medical College of Rzeszow University, 35-959 Rzeszow, Poland; r.s.filip@wp.pl; 4 Department of Gastroenterology with IBD Unit of Clinical Hospital 2 im. Św. Jadwigi Królowej, 35-301 Rzeszow, Poland

**Keywords:** endoscopic laboratory, microbiological cleanliness, pathogenic microorganisms, nosocomial infections, environmental health

## Abstract

The increasing number of endoscopic procedures performed and their increasing invasiveness mean that endoscopy of the gastrointestinal tract is associated with the risk of transmitting pathogenic microorganisms through infected equipment or contact with other patients and medical staff. In order to ensure protection of the health of both patients and medical staff, endoscopy laboratories should meet high hygiene standards. The results of tests of the microbiological cleanliness of surfaces and equipment of an endoscopic examination laboratory performed in the period from January to December 2019 at the Provincial Clinical Hospital No. 2 in Rzeszow were assessed retrospectively. Samples for testing were collected by swabbing from places where microbiological contamination was the most likely and cleaning was the most difficult. In the analyzed period, a total of 86 samples were collected for microbiological tests, of which positive results accounted for 6.9%. Positive results were obtained mainly from swabs collected from wet surfaces (66.7%). Most of the isolated microorganisms were Gram-negative bacteria (66.7% of all positive tests) and they were: *Acinetobacter junii, Ralstonia pickettii*, and *Achromobacter denitrificans*. The condition of the microbiological cleanliness of the surfaces and equipment of the endoscopic examination laboratory was satisfactory. A very low level of microbiological contamination of the tested items indicates occasional shortcomings in the decontamination processes. Since microorganisms isolated from the collected samples may be the cause of infection in patients and medical personnel, it is necessary to verify the decontamination procedures applied and to continue periodic microbiological monitoring of their effectiveness.

## 1. Introduction

Endoscopic examinations of the gastrointestinal tract are diagnostic and therapeutic methods commonly used in gastroenterology and surgery, and should be carried out in modern and well-equipped laboratories [1].

The growing number of endoscopic procedures and their increasing invasiveness mean that endoscopy of the gastrointestinal tract is associated with the risk of transmitting pathogenic microorganisms through infected equipment or contact with other patients and medical personnel. In order to ensure protection of the health of both patients and medical staff, endoscopy laboratories (gastrointestinal endoscopy units) should meet high hygiene standards [2,3,4].

The spread of infections in the endoscopic laboratory can be limited, among others, by strict compliance with hygiene rules among employees, systematic monitoring of the sanitary and hygiene conditions and increasing the intensity of monitoring of the microbiological cleanliness of rooms and equipment [5]. Infections can also be reduced through the selection of appropriate detergents and disinfectants [6]. The spread of infections is also reduced by replacing reusable endoscopic accessories with disposable equipment. An example of systemic solutions in this area is the project “Development of single-use endoscopic instrument” (DUET), an EU-funded project focused on the development of disposable endoscopes to reduce the risk of cross-contamination and nosocomial infections. It demonstrated the possibility of designing cost-effective endoscopic equipment that can be safely disposed of after use and, in the future, recycled [7].

Available medical literature does not include any reports on the topic described.

### Aim

Qualitative assessment of the microbiological cleanliness of surfaces and equipment in an endoscopic examination laboratory.

## 2. Materials and Methods

The results of tests of the microbiological cleanliness of surfaces and equipment of an endoscopic examination laboratory performed in the period from January to December in 2019 at the Provincial Clinical Hospital No. 2 in Rzeszow were subject to retrospective evaluation. Samples for testing were collected once a quarter by swabbing from places where microbiological contamination was the most likely and cleaning was the most difficult. Samples were collected with sterile dry swabs tipped with a viscose swab, which were placed in tubes with Amies Transport Medium after the swabs were collected.

Monitoring of microbiological cleanliness did not include standard medical instruments. The rules for the decontamination of endoscopic equipment are commonly used and described in the literature [8,9,10,11,12,13].

In accordance with the methodology in force, the collected material was incubated in a broth enriched at 37 °C for 24 h. In the case of propagation of positive cultures in the broth, it was streaked onto solid media: 5% sheep blood agar and MacConkey. The blood agar and MacConkey plates were incubated for 24 h at 37 °C. In the case of growth on the solid media, microorganisms were identified using the VITEK MS automated mass spectrometer (BIOMERIEUX, Marcy-l’Étoile, France), using MALDI-TOF technology [14,15,16]. MS enables the reliable identification of human pathogens as well as zoonotic and environmental microorganisms [17]. This technique, based on Matrix Assisted Laser Desorption Ionization Time-of-Flight (MALDI-TOF), uses an extensive microbial database of bacteria and fungi [18,19,20]. Due to the simplicity of implementation and the automation of the diagnostic process, MS allows for a significant reduction in the examination time and reliable identification even with a small number of microbial cells. Quick and reliable results enable the implementation of appropriate epidemiological surveillance procedures [21,22,23,24,25,26].

The isolated and identified microorganisms were assessed for drug susceptibility using the radial diffusion method or the automated VITEK2 system (BIOMERIEUX) [27,28].

## 3. Results

In the analyzed period, a total of 56 swabs from dry surfaces were collected, including: keyboard, telephone, remote control, buttons of medical equipment, glasses, internal surfaces of the cabinet for storing endoscopic equipment, surface of the treatment table, and transport trolley, and 30 swabs were collected from wet surfaces, i.e., washbasin faucet knobs, soap dispenser lever, distilled water containers, and wash basin.

Swabs from which potentially pathogenic microorganisms were cultured made up 6.9% of all the samples taken. Gram-negative bacteria, i.e., *Acinetobacter junii, Ralstonia pickettii* and *Achromobacter denitrificans*, were present in four cases (66.7%). From two samples (33.3%), bacteria from the Gram-positive group, i.e., *Staphylococcus aureus*, were grown—Table 1.

The same bacterial susceptibility profile was discovered in two strains of *Achromobacter denitrificans*, though they were found in material collected from different containers for distilled water at the same time. This fact may indicate a common origin.

The evaluation of the obtained data did not reveal any seasonal variability in the scope of the identified microbiological contamination, while the analysis of the profile of bacterial resistance to antibiotics demonstrates a high level of sensitivity of the cultured microorganisms to commonly used antibiotics—Table 1.

Positive results of microbiological tests were obtained in the case of four samples taken from wet surfaces, i.e., from containers for distilled water (66.7%) and from dry surfaces, i.e., washing station buttons and a computer keyboard (33.3%)—Table 2.

## 4. Discussion

Each year, many millions of people around the world suffer from healthcare-associated infections, which often aggravate the patient’s symptoms and prolong hospitalization. They can also be the cause of permanent disability and, in many cases, lead to the patient’s death. In addition, healthcare-associated infections increase financial costs for patients and hospitals, and prolonged hospitalization and the increased need for specialist medical care place a significant burden on the healthcare system [29,30].

Patients who are particularly susceptible to colonization and microbial infection include those suffering from immunocompromising diseases. Infections can be reduced by scrupulous adherence to hygiene procedures, following guidelines for the control of nosocomial infections, and shortening the patient’s stay in hospital [29,31].

In this context, proper sanitary and hygiene conditions in a hospital are essential to reducing the risk of infection.

The endoscopic laboratory is a place that requires special attention to the quality of medical equipment and the safety of the procedures performed. Due to the type of treatments carried out there and the possibility of infection, effective decontamination is necessary to ensure a high level of microbiological cleanliness of equipment and rooms [1,3,4].

In endoscopic examination laboratories, it is also necessary to carry out systematic microbiological supervision regarding the effectiveness of the hygiene and sanitary procedures performed. Guidelines in this regard are in force in many countries around the world [32,33,34,35,36,37,38,39,40,41].

Our own research has shown that activities in the field of microbiological supervision of decontamination processes are undertaken at the Provincial Clinical Hospital No. 2 in Rzeszow, in the form of the periodic assessment of the microbiological cleanliness of the environment of the Endoscopic Examination Laboratory, conducted by the employees of the Clinical Department of Microbiology. As part of this monitoring, in 2019, 6.9% of swabs taken from the surfaces and equipment in the laboratory were found to be positive. Among the isolated microorganisms, *Acinetobacter junii, Ralstonia pickettii, Achromobacter denitrificans* and *Staphylococcus aureus* were found. All of these microorganisms pose a serious risk in a hospital setting.

*Acinetobacter* bacillus species occur naturally in soil and water. In the difficult conditions of the hospital environment, they live on wet and dry surfaces. These bacteria can often be found on the surfaces of apparatus, medical equipment, general equipment and on the hands of personnel. *Acinetobacter* infection may occur, especially in immunocompromised patients [42].

*Ralstonia pickettii* aerobic bacilli are often found in wet environments such as soil, rivers, lakes and, as oligotrophic organisms, can survive in environments with very low nutrient concentrations. This microorganism is also found in hospitals around the world [43]. *Ralstonia pickettii* is a pathogen particularly dangerous in immunocompromised patients and those treated in the ICU [16,44].

This bacterium is characterized by a very high resistance to water treatment processes in water supply systems and to the action of disinfectants (including chlorhexidine). In the literature, cases of infection in patients as a result of contact with contaminated water, saline or medications have been reported. Aqueous solutions of medicinal products may become contaminated because *Ralstonia pickettii* has the ability to pass through 0.45 and 0.2 µm filters, which are often used to sterilize liquid substances [15,43,44,45,46].

*Ralstonia pickettii* bacilli have low nutritional requirements and can survive for a long time in water (including distilled water) and soil. The virulence of these bacteria is manifested by their ability to produce toxins and biofilm, which allows the cells to live in plastic pipes, implants and in medical devices such as endoscopes [16,43,45,47,48].

In patients, *Ralstonia pickettii* can cause asymptomatic infections, as well as infections associated with bacteremia, severe sepsis and septic shock. *Ralstonia pickettii* is an etiological factor, among others, of pneumonia, endocarditis, peritonitis and infections associated with the use of venous catheters [44,45].

*Ralstonia pickettii* bacteremia should always prompt a search for sources of infection in contaminated medical products and fluids [45].

*Achromobacter denitrificans* bacillus is a Gram-negative, obligatory aerobic bacterium that often lives in soil, human intestines and in the aquatic environment, e.g., in contaminated intravenous fluids or water in air humidifiers [27,49,50,51]. In hospital conditions, infections caused by this microorganism are associated with the use of humidifiers and incubators. Most infections develop during a hospital stay and are asymptomatic. Symptomatic infection may appear as endocarditis, meningitis, pneumonia, peritonitis, conjunctivitis, osteomyelitis, and urinary tract or wound infection. Invasive *Achromobacter denitrificans* infections can be fatal in immunocompromised individuals [49,50,51].

The mortality rate for infections caused by *Achromobacter* species ranges from 3% in primary bacteremia or catheterization-related infections to 80% in severe neonatal infections. Mortality is also higher in patients over 65 years of age, in patients with neutropenia and in polymicrobial infections [49,51].

*Staphylococcus aureus* is another pathogen found in samples taken from dry surfaces in the endoscopic laboratory.

Data from the literature show that these bacteria are widely distributed in the hospital environment, among patients and medical staff. *Staphylococcus aureus* colonizes the surface of the skin and mucous membranes extremely easily, especially damaged ones. The high resistance of *Staphylococcus aureus* to environmental conditions allows it to survive longer outside the host organism, including on contaminated surfaces of medical equipment and apparatus [52].

The most common and typical infections caused by *Staphylococcus aureus* in hospitalized patients are surgical wound infections, pneumonia, meningitis after cardiac surgery and infections in newborns in intensive care units [52].

In the gastrointestinal endoscopy unit, about 3000 examinations are carried out annually and, therefore, also the same number of decontamination processes. As a result of the qualitative tests of the microbiological cleanliness of non-medical equipment, the presence of microorganisms was found in 6 samples out of 86 collected during the year. No alarm factors were found, so the risk of infection with such pathogens in the analyzed period was close to zero. In our research, we rely on the Polish legal regulations currently in force [53].

Compliance with the standards regarding the decontamination process is an important criterion for the quality of work in the endoscopic examination laboratory and guarantees safety in the scope of the diagnostic and therapeutic services provided there.

Microbiological monitoring of the laboratory environment after decontamination is useful in detecting and eliminating errors made during the processes of cleaning and disinfecting rooms and equipment. Systematic microbiological supervision guarantees a reliable method of monitoring compliance with the applicable hygiene and sanitary standards.

The limitation of our study was the fact that samples were not taken before and after the decontamination process, which made it impossible to obtain comparative data.

No studies of a similar subject have been found in the available medical literature.

## 5. Conclusions

The condition of the microbiological cleanliness of surfaces and equipment of the endoscopic examination laboratory was satisfactory. The very low level of microbiological contamination of the tested items indicates occasional shortcomings in the decontamination processes. The presented test results, despite the small number of positive cultures found, indicate that microbiological monitoring of endoscopic laboratories and their facilities should be carried out periodically, not less frequently than once per quarter, and the data trended. When determining the sampling frequency, the current epidemic situation in the hospital and in a given geographical region should be taken into account. The results of such inspections should be compared with the results of microbiological tests carried out in other organizational units of the hospital as well as among staff. Since microorganisms isolated from the collected samples may cause infection in patients and medical staff, in the case of systematically repeated positive microbiological test results consideration should be given to reviewing the decontamination procedures applied and training of the laboratory staff, as well as intensifying periodic microbiological monitoring of their effectiveness.

## Figures and Tables

**Table 1:** Swabs taken from surfaces and equipment of an endoscopic examination laboratory in 2019 and cultured microorganisms.

Quarter	Number of Samples Collected *n*	Positive Results *n*/%	Cultivated Microorganisms	Susceptibility of the Cultured Microorganisms *
I	20	0/0%	-	-
II	20	2/10.0%	*Acinetobacter junii*	SXT(s), GM(s), CIP(s), CAZ(s), TZP(s)
			*Staphylococcus aureus*	SXT(s), CM(s), E(s), P(s), OX(s)
III	20	2/10.0%	*Achromobacter denitrificans*	IPM(s), CIP(s), GM(s), PIP(s)
			*Achromobacter denitrificans*	IPM(s), CIP(s), GM(s), PIP(s)
IV	26	2/7.7%	*Staphylococcus aureus*	SXT(s), CM(s),E(s), OX(s)
			*Ralstonia pickettii*	CTX(s), CIP(s), SXT(s), IPM(s)
Total	86	6/6.9%	-	-

* Legend: s—sensitive; CAZ—Ceftazidime; CTX—Cefotaxime; IPM—Imipenem; PIP—Piperacillin; CIP—Ciprofloxacin; E—Erythromycin; OX—Oxacillin; SXT—Trimethoprim/Sulfamethoxazole; CM—Clindamycin; GM—Gentamicin; P—Penicillin; TZP—Piperacillin/Tazobactam.

**Table 2:** Type of surfaces with the presence of potentially pathogenic microorganisms.

Wet Surfaces			Dry Surfaces		
Surface	Positive Microbiological Results *n*/% of All Positive Results	Cultivated Microorganisms	Surface	Positive Microbiological Results *n*/% of All Positive Results	Cultivated Microorganisms
Distilled water containers	4/66.7%	*Acinetobacter junii* *Achromobacter denitrificans* *Achromobacter denitrificans* *Ralstonia pickettii*	Washing station buttons	1/16.65%	*Staphylococcus aureus*
			Computer keyboard	1/16.65%	*Staphylococcus aureus*
Total	4/66.7%	-	-	2/33.3%	-

## Data Availability

Data sharing not applicable.
